# Interaction between *IRF6* and *TGFA* Genes Contribute to the Risk of Nonsyndromic Cleft Lip/Palate

**DOI:** 10.1371/journal.pone.0045441

**Published:** 2012-09-20

**Authors:** Ariadne Letra, Walid Fakhouri, Renata F. Fonseca, Renato Menezes, Inga Kempa, Joanne L. Prasad, Toby G. McHenry, Andrew C. Lidral, Lina Moreno, Jeffrey C. Murray, Sandra Daack-Hirsch, Mary L. Marazita, Eduardo E. Castilla, Baiba Lace, Ieda M. Orioli, Jose M. Granjeiro, Brian C. Schutte, Alexandre R. Vieira

**Affiliations:** 1 Department of Oral Biology, University of Pittsburgh, School of Dental Medicine, Pittsburgh, PA, USA; 2 Department of Pediatric Dentistry, University of Pittsburgh, School of Dental Medicine, Pittsburgh, PA, USA; 3 Department of Diagnostic Sciences, University of Pittsburgh, School of Dental Medicine, Pittsburgh, PA, USA; 4 Center for Craniofacial and Dental Genetics, University of Pittsburgh, School of Dental Medicine, Pittsburgh, PA, USA; 5 Department of Microbiology and Molecular Genetics, Michigan State University, East Lansing, MI, USA; 6 Department of Genetics, Institute of Biology, Center of Health Sciences; Federal University of Rio de Janeiro; Rio de Janeiro, RJ, Brazil; 7 Latvian Biomedical Research and Study Centre, Riga, Latvia; 8 Department of Biology and Microbiology, Riga Stradins University, Riga, Latvia; 9 Department of Orthodontics, College of Dentistry, University of Iowa, Iowa City, IA, USA; 10 Department of Pediatrics, Roy J. and Lucille A. Carver College of Medicine, University of Iowa, Iowa City, IA, USA; 11 Department of Human Genetics, Graduate School of Public Health, University of Pittsburgh, Pittsburgh, PA, USA; 12 Clinical and Translational Science Institute, University of Pittsburgh, Pittsburgh, PA, USA; 13 ECLAMC (Latin American Collaborative Study of Congenital Malformations) at CEMIC (Center of Medical Education and Clinical Research “Norberto Quirno”), Buenos Aires, Argentina; 14 CONICET (National Research Council of Argentina), Buenos Aires, Argentina; 15 iNaGeMP-CNPq (National Institute of Population Medical Genetics), Porto Alegre, RS, Brazil; 16 Department of Cell and Molecular Biology, Fluminense Federal University, Niterói, RJ, Brazil and INMETRO, Duque de Caxias, RJ, Brazil; University of Miami, United States of America

## Abstract

Previous evidence from tooth agenesis studies suggested *IRF6* and *TGFA* interact. Since tooth agenesis is commonly found in individuals with cleft lip/palate (CL/P), we used four large cohorts to evaluate if *IRF6* and *TGFA* interaction contributes to CL/P. Markers within and flanking *IRF6* and *TGFA* genes were tested using Taqman or SYBR green chemistries for case-control analyses in 1,000 Brazilian individuals. We looked for evidence of gene-gene interaction between *IRF6* and *TGFA* by testing if markers associated with CL/P were overtransmitted together in the case-control Brazilian dataset and in the additional family datasets. Genotypes for an additional 142 case-parent trios from South America drawn from the Latin American Collaborative Study of Congenital Malformations (ECLAMC), 154 cases from Latvia, and 8,717 individuals from several cohorts were available for replication of tests for interaction. *Tgfa* and *Irf6* expression at critical stages during palatogenesis was analyzed in wild type and *Irf6* knockout mice. Markers in and near *IRF6* and *TGFA* were associated with CL/P in the Brazilian cohort (p<10^−6^). *IRF6* was also associated with cleft palate (CP) with impaction of permanent teeth (p<10^−6^). Statistical evidence of interaction between *IRF6* and *TGFA* was found in all data sets (p = 0.013 for Brazilians; p = 0.046 for ECLAMC; p = 10^−6^ for Latvians, and p = 0.003 for the 8,717 individuals). *Tgfa* was not expressed in the palatal tissues of *Irf6* knockout mice. *IRF6* and *TGFA* contribute to subsets of CL/P with specific dental anomalies. Moreover, this potential *IRF6-TGFA* interaction may account for as much as 1% to 10% of CL/P cases. The *Irf6*-knockout model further supports the evidence of *IRF6-TGFA* interaction found in humans.

## Introduction

Oral-facial clefts are common birth defects with an incidence of 1–2 in 1000 live births, thus comprising almost one-half of all craniofacial anomalies. They impose adverse health, social, and economic implications for the affected individuals and their families [1]. Although the mortality and morbidity of an infant born with a cleft lip and or a cleft palate has improved greatly in the last century, it is still elevated for infants born with multiple additional anomalies. Among the consequences of being born with clefts are shorter life span and increased risk for all major causes of death when compared to individuals without clefts [2].

Cleft lip with or without cleft palate (herein called cleft lip/palate) can be classified as nonsyndromic or syndromic based on the presence of other associated congenital defects. Approximately 20–50% of all cleft cases are associated with one of more than 400 syndromes [3]. Syndromic forms usually present Mendelian inheritance patterns, which allow identification of causal genes. Nonsyndromic cleft lip/palate however, is considered a genetically complex trait with no clearly recognizable inheritance pattern [4].

Identifying the key genes responsible for the genesis of cleft lip/palate is fundamental for elucidating the pathogenetic mechanisms and developing measures for its management and prevention. Studies have estimated that 3–14 genes interacting multiplicatively may be involved in the etiology of cleft lip/palate [5], and a variety of genes have been associated and suggested to play a role in the genetic susceptibility to cleft lip/palate [4].

To date, the most consistent finding for the genetic etiology of nonsyndromic cleft lip/palate has been the association of the interferon regulatory factor 6 (*IRF6*) gene at 1q32 [6], previously identified as etiologic for Van der Woude syndrome which includes cleft lip/palate as part of the clinical spectrum [7]. A particularly strong overtransmission of the ancestral allele V at a V274I polymorphism (rs2235371) was detected in individuals of Asian and South American ancestry from 8,003 individuals representing ten distinct populations. Attributable risk calculations suggested *IRF6* could contribute to as much as 12% of all cleft cases [6]. Intriguingly, additional studies with different populations have consistently shown positive association between markers in *IRF6* and cleft lip/palate [8]–[22]. The frequency of the V274I risk allele is over 97% in European and African populations making it an unlikely candidate for the etiological mutation.

The association of the transforming growth factor alpha (*TGFA*) gene at 2p13 with cleft lip/palate has also rendered intriguing results. *TGFA* was the first gene associated to nonsyndromic cleft lip/palate in a case-control study [23]. Several studies followed with rather discrepant results; in turn, comparison among studies with *TGFA* has been somewhat difficult due to unaccounted variations in study design, markers tested and percentages of patients with positive family history [24]. Meta-analytic approaches [25], [26] concluded that *TGFA* plays a small but significant role in cleft lip/palate with odds ratios indicating a modest effect size. Instead of an effector gene, *TGFA* has been regarded as a]modifier to the clefting phenotype [24].

Evidence from tooth agenesis studies suggested that *IRF6* and *TGFA* genes may interact [27], [28]. Tooth agenesis is a common congenital anomaly where one or more permanent teeth are absent and is a frequent observation in individuals with cleft lip/palate. Therefore interaction between *IRF6* and *TGFA* in tooth agenesis may also be relevant to cleft lip/palate. Since tooth agenesis is commonly found in individuals with cleft lip/palate, we used three large samples of cleft cases to test for interaction between *IRF6* and *TGFA* in the etiology of the cleft phenotype.

## Results

### Results of Case-control Comparisons

Tables 1 and 2 summarize the studied Brazilian samples and genetic markers. There were no evidences of deviation from Hardy-Weinberg equilibrium for any of the markers in cases and controls (data not shown). Table 3 summarizes the linkage disequilibrium relationships of the markers studied.

**Table 1:** Baseline clinical characteristics of the Brazilian population.

Populations	Cleft		Control	
	N	%	N	%
**Age**				
*Range*	4–59	–	4–94	–
*Mean*	17.32	–	36.8	–
**Gender**				
*Males*	302	60	165	33
*Females*	198	40	335	67
**Race**				
*Caucasian*	406	82	285	58.2
*African*	79	16	38	7.8
*Asian*	9	2	167	34
*Unknown*	6	16	10	18

**Table 2:** Details of the SNPs investigated in this study.

SNP marker	Base Position^a^	Approximate Llocation	Function	BaseChange	Average Heterozygozity	Type of Assay
*IRF6*						
rs4844880	207,937,539	90 kb 3′ of IRF6	intron	**AT**	0.488+/−0.075	Taqman OD^b^
rs2235371 (V274I)	208,030,703	In IRF6	missense	**CT**	0.247+/−0.250	Taqman OD^b^
rs2013162	208,035,307	In IRF6	coding-synonymous	**AC**	0.478+/−0.102	Taqman OD^b^
rs861019	208,042,009	In IRF6	5′UTR	**AG**	0.474+/−0.111	Taqman OD^b^
rs2073487	208,043,269	In IRF6	intron	**CT**	0.479+/−0.099	Taqman OD^b^
rs658860	208,057,172	11 kb 5′ of IRF6	unknown	**CT**	0.290+/−0.247	Taqman OD^b^
*TGFA*						
rs1058213 (C3827T)	70,530,971	In TGFA	3′UTR	**CT**	0.286+/−0.247	Kinetic PCR
rs2166975 (C3296T)	70,531,502	In TGFA	coding-synonymous	**GA**	0.340+/−0.233	Kinetic PCR
rs930655	70,537,959	In TGFA	intron	**AG**	0.438+/−0.166	Taqman OD^b^
rs1523305	70,552,364	In TGFA	intron	**CT**	0.497+/−0.038	Taqman OD^b^
rs2902345	70,570,107	In TGFA	intron	**CT**	0.471+/−0.118	Taqman OD^b^
rs377122	70,620,533	In TGFA	intron	**CT**	0.485+/−0.085	Taqman OD^b^

a According to the USCS Genome Browser on Human March 2006 Assembly (hg18).

b Assay-on-demand.

**Table 3:** Results of linkage disequilibrium analyses for the investigated markers in the Brazilian Caucasian population (406 cases and 285 controls).

Markers	rs4844880	rs2235371	rs2013162	rs861019	rs2073487	rs658860
*IRF6*						
rs4844880	–	0.057	0.009	0.011	0.009	0.006
rs2235371	0.538	–	0.091	0.005	0.093	0.007
rs2013162	0.221	0.858	–	0.070	0.986	0.090
rs861019	0.190	0.309	0.385	–	0.066	0.030
rs2073487	0.223	0.860	1.000	0.375	–	0.095
rs658860	0.097	0.677	0.873	0.404	0.903	–
	rs1058213	rs2166975	rs930655	rs1523305	rs2902345	rs377122
*TGFA*						
rs1058213	–	0.043	0.017	0.024	0.018	0.0001
rs2166975	0.434	–	0.032	0.036	0.031	0.002
rs930655	0.536	0.450	–	0.223	0.232	0.008
rs1523305	0.742	0.413	0.536	–	0.741	0.018
rs2902345	0.712	0.420	0.494	0.954	–	0.015
rs377122	0.065	0.074	0.092	0.160	0.160	–

r^2^ is above the diagonal; D’ is below the diagonal.

Table 4 summarizes the results of the association analysis obtained for Brazilian Caucasian cases (N = 406) and controls (N = 285) for each marker studied, according to each cleft subphenotype. When comparing Brazilian cleft cases with controls, we observed an association between the intronic marker rs2902345 with cleft lip/palate (P<0.001). For *IRF6*, we found significant association between the V274I polymorphism (rs2235371) with complete left cleft lip/palate (P<0.001). An intronic marker in *IRF6* (rs2073487) also showed a trend for association with complete left cleft lip/palate (P = 0.0009).

**Table 4:** Summary of the association analysis in Brazilian Caucasians according to each cleft subphenotype.

Subphenotype	n	*TGFA*											
		rs1058213genotypep-value	rs1058213allelep-value	rs2166975genotypep-value	rs2166975allelep-value	rs930655genotypep-value	rs930655allelep-value	rs1523305genotypep-value	rs1523305allelep-value	rs2902345genotypep-value	rs2902345allelep-value	rs377122genotypep-value	rs377122genotypep-value
CP	53	1.0	0.91	0.44	0.18	0.37	0.45	0.32	0.28	0.31	0.43	0.17	1.0
CL/P	324	0.68	0.86	0.34	0.09	0.45	0.81	0.71	0.63	**0.00001**	0.02	0.007	0.68
Complete	237	0.39	0.51	0.10	0.01	0.41	0.68	0.64	0.43	0.60	0.44	0.04	0.39
Incomplete	86	0.76	0.95	0.78	0.47	0.30	0.13	0.77	0.60	0.96	0.95	0.007	0.76
CL/P Unilateral	200	0.37	0.95	0.21	0.06	0.16	0.85	0.59	0.99	0.52	0.55	0.005	0.37
Complete	136	0.19	0.81	0.01	0.001	0.10	0.33	0.48	0.82	0.37	0.54	0.01	0.19
Incomplete	64	0.78	0.62	0.31	0.09	0.35	0.24	0.90	0.70	0.97	0.80	0.007	0.78
CL/P Right	60	0.37	0.06	0.92	0.64	0.15	0.46	0.75	0.47	0.62	0.41	0.23	0.37
Complete	43	0.08	0.001	0.50	0.19	0.40	0.36	0.54	0.32	0.73	0.46	0.46	0.08
Incomplete	17	0.96	0.50	0.49	0.18	0.16	0.97	0.54	0.80	0.80	0.65	0.30	0.96
CL/P Left	140	0.19	0.37	0.13	0.04	0.39	0.83	0.35	0.67	0.30	0.79	0.006	0.19
Complete	93	0.05	0.11	0.008	0.001	0.17	0.51	0.31	0.35	0.18	0.75	0.01	0.05
Incomplete	47	0.84	0.87	0.55	0.23	0.39	0.17	0.57	0.56	0.96	0.96	0.01	0.84
CL/P Bilateral	123	0.59	0.36	0.40	0.34	0.69	0.41	0.67	0.40	0.90	0.67	0.18	0.59
Complete	101	0.44	0.13	0.46	0.64	0.89	0.64	0.44	0.26	0.76	0.53	0.54	0.44
Incomplete	22	0.72	0.31	0.42	0.14	0.09	0.03	0.73	0.56	0.79	0.82	0.06	0.72
CL/P + CP	393	0.66	0.51	0.28	0.07	0.82	0.88	0.80	0.53	0.64	0.48	0.09	0.66

Subphenotype	n	*IRF6*											
		rs4844880ggenotypep-value	rs4844880allelep-value	rs2235371genotypep-value	rs2235371allelep-value	rs2013162genotypep-value	rs2013162allelep-value	rs861019genotypep-value	rs861019allelep-value	rs2073487genotypep-value	rs2073487allelep-value	rs658860genotypep-value	rs658860allelep-value
CP	53	0.91	0.85	0.02	0.17	0.17	0.05	0.06	0.03	0.20	0.07	0.75	0.84
CL/P	324	0.73	0.70	0.32	0.15	0.02	0.01	0.45	0.21	0.008	0.01	0.24	0.09
Complete	237	0.39	0.44	0.65	0.46	0.13	0.08	0.49	0.24	0.06	0.04	0.50	0.30
Incomplete	86	0.09	0.02	0.14	0.003	0.02	0.005	0.42	0.40	0.03	0.03	0.25	0.09
CL/P Unilateral	200	0.58	0.32	0.22	0.18	0.03	0.04	0.26	0.22	0.01	0.06	0.46	0.43
Complete	136	0.58	0.32	0.22	0.18	0.03	0.04	0.26	0.22	0.01	0.06	0.46	0.43
Incomplete	64	0.51	0.59	0.49	0.66	0.06	0.15	0.51	0.34	0.05	0.10	0.66	0.64
CL/P Right	60	0.03	0.008	0.38	0.15	0.19	0.06	0.29	0.30	0.10	0.23	0.48	0.37
Complete	43	0.13	0.04	0.39	0.50	0.36	0.28	0.43	0.39	0.04	0.62	0.26	0.30
Incomplete	17	0.005	0.001	0.77	0.46	0.68	0.42	0.97	0.88	0.05	0.79	0.48	0.92
CL/P Left	140	0.45	0.91	0.64	0.19	0.56	0.34	0.69	0.77	0.55	0.33	0.03	0.89
Complete	93	0.07	0.13	**0.0002**	**0.00001**	0.009	0.001	0.10	0.10	0.005	0.0009	0.03	0.01
Incomplete	47	0.43	0.23	0.49	0.23	0.21	0.08	0.14	0.45	0.18	0.06	0.14	0.05
CL/P Bilateral	123	0.59	0.55	0.39	0.18	0.15	0.04	0.35	0.44	0.06	0.01	0.17	0.06
Complete	101	0.57	0.46	0.64	0.44	0.40	0.19	0.25	0.36	0.28	0.10	0.43	0.19
Incomplete	22	0.82	0.84	0.23	0.08	0.04	0.008	0.99	0.92	0.03	0.007	0.06	0.04
CL/P + CP	393	0.69	0.57	0.24	0.37	0.31	0.16	0.77	0.46	0.17	0.14	0.51	0.26

Genotype p-values are listed first, followed by allele p-values.

* P≤0.0002 indicates statistical difference (in bold) in comparison to controls (n = 285). Unknown cleft types (n = 16) were not considered for analyses.

† For both genotype and allele.

We also compared cleft subphenotypes with tooth agenesis and other dental anomalies and controls. Table 5 summarizes the results of the association analysis obtained in the Brazilian cases (N = 406) and controls (N = 285) for *TGFA* and *IRF6* markers, according to each cleft subphenotype with dental anomalies. Although genotype/allele frequencies did not significantly differ between cases presenting with tooth agenesis and controls, we found an association between the V274I marker in *IRF6* and cleft palate in the presence of impaction of permanent teeth (P<0.0001).

**Table 5:** Summary of the association analysis in Brazilian Caucasians according to each cleft subphenotype with dental anomalies.

Cleft subphenotypes with dental anomalies	n	*TGFA*															
		rs1058213genotypep-value	rs1058213genotypep-value	rs2166975genotypep-value	rs2166975allelep-value	rs930655genotypep-value	rs930655allelep-value	rs1523305genotypep-value	rs1523305allelep-value	rs2902345genotypep-value		rs2902345allelep-value		rs377122genotypep-value		rs377122genotypep-value	
CP with agenesis	12	0.90	0.67	0.06	0.04	0.15	0.07	0.04	0.08	0.06		0.09		0.41		0.21	
CP with impaction of permanent teeth	2	–	–	–	–	–	0.006	–	0.01	–		0.008		–		0.01	
Right CL/P with agenesis	24	0.24	0.02	0.94	0.69	0.78	0.57	0.97	0.83	0.32		0.16		0.95		0.60	
Left CL/P with agenesis	36	0.01	0.48	0.06	0.03	0.38	0.20	0.51	0.72	0.21		0.16		0.38		0.29	
Bilateral CL/P with agenesis	34	0.88	0.97	0.29	0.17	0.73	0.96	0.81	0.88	0.09		0.03		0.71		0.50	
Unsuccessful bilateral CL/P*	26	0.02	0.31	0.93	0.96	0.62	0.94	0.20	0.60	0.87		0.97		0.33		0.26	
**Cleft subphenotypes with dental anomalies**	**n**	***IRF6***															
		**rs4844880** **genotype** **p-value**	**rs4844880** **genotype** **p-value**	**rs2235371genotype** **p-value**	**rs2235371allele** **p-value**	**rs2013162genotype** **p-value**	**rs2013162** **allele** **p-value**	**rs861019** **genotype** **p-value**	**rs861019** **allele** **p-value**	**rs2073487** **genotype** **p-value**		**rs2073487** **allele** **p-value**		**rs658860genotype** **p-value**	**rs658860genotype** **p-value**		
CP with agenesis	12	0.47	0.87	0.16	0.12	0.93	0.75	0.24	0.90	0.76	0.53		0.001		0.01		
CP with impaction of permanent teeth	2	0.79	0.88	**0.00001**	**0.00001**	0.002	0.003	–	–	–	–		–		–		
Right CL/P with agenesis	24	0.27	0.58	0.20	0.81	0.94	0.83	0.67	0.69	0.93	0.79		0.30		0.41		
Left CL/P with agenesis	36	0.75	0.78	0.56	0.32	0.54	0.36	0.10	0.25	0.50	0.33		0.19		0.39		
Bilateral CL/P with agenesis	34	0.57	0.28	0.78	0.74	0.57	0.34	0.29	0.35	0.44	0.23		0.80		0.52		
Unsuccessful bilateral CL/P*	26	0.54	0.33	0.03	0.75	0.46	0.53	0.86	0.70	0.47	0.57		0.62		0.54		

* Unilateral CL/P with agenesis or microdontia of the maxillary lateral incisor on the noncleft side.

** P≤0.0008 indicates significant difference (in bold).

† For both genotype and allele.

### Results of Attributable Fraction Calculations for *IRF6-TGFA* Interaction

We calculated the attributable fraction (AF) for the high-risk alleles at *IRF6* V274I and *TGFA* C3827T (P = 0.03) for the Brazilian sample, and the estimated contribution of the interaction between these two genes in this population was found to be approximately 1% (Table 6).

**Table 6:** Results for interaction of *TGFA* C3827T and *IRF6* V274I marker alleles in the Brazilian Caucasian cases (N = 406) and controls (N = 285).

*TGFA* rs1058213 (C3827T)		*IRF6* rs2235371 (V274I)		P-value*
		**allele 1 (C)**	**allele 2 (T)**	
**allele 1 (C)**	cases	355	3	0.49
	controls	256	1	
**allele 2 (T)**	cases	16	0	0
	controls	15	0	
		**allele 1 (C)**	**allele 2 (T)**	
**allele 1 (C)**	cases	327	31	0.35
	controls	229	28	
**allele 2 (T)**	cases	13	3	0.08
	controls	15	0	
		**allele 1 (C)**	**allele 2 (T)**	
**allele 1 (C)**	cases	351	3	0.49
	controls	255	1	
**allele 2 (T)**	cases	20	0	0
	controls	16	0	
		**allele 1 (C)**	**allele 2 (T)**	
**allele 1 (C)**	cases	325	29	0.25
	controls	228	28	
**allele 2 (T)**	cases	15	5	**0.03**
				
	controls	16	0	

* Mantel-Haenszel test; p≤0.05 indicates statistical difference (in bold).

We also tested for *IRF6-TGFA* interaction in the ECLAMC samples by observing the transmission of the high-risk alleles at *IRF6* V274I and *TGFA* C3827T in the 142 case-parent trios and detected significant overtransmission of these alleles to the affect child (P = 0.001). The attributable fraction for these samples (AF = 0.04) suggests this interaction may account for ∼4% of the cases of cleft lip/palate in this particular population. Genotypes used for these calculations are included as Supplemental Material (Table S1).

Analysis of genotypes in additional 7,047 people from seven distinct populations provides suggestive evidence of interaction between two *TGFA* markers (rs3732253 and rs377122) with a polymorphism in *IRF6* (rs2013162) among Caucasians (P = 0.02) and Asians (P = 0.03) (although these p-values are nominal and would not be significant under strict Bonferroni correction). Analysis of the pooled samples indicates statistical interaction between a marker at *TGFA* (rs1807968) and another marker at *IRF6* (rs2013162) in the cleft lip only group (P = 0.003) (Table 7). Attributable fraction calculations (AF = 0.10) further suggest ∼10% of cleft lip cases may be attributed to such interaction in the general population.

**Table 7:** Results of interaction analyses of associated *TGFA* and *IRF6* marker alleles in the family dataset (861 simplex and multiplex families) comprising 7047 people stratified by population origin and cleft types.

SNPs		All Samples			ASIA		PHILIPPINES		CAUCASIANS		COLOMBIA		INDIA	
*TGFA*	*IRF6*	Initial FBAT p-value	Corrected p-value	Nominal p-value	Corrected p-value	Nominal p-value	Corrected p-value	Nominal p-value	Corrected p-value	Nominal p-value	Corrected p-value	Nominal p-value	Corrected p-value	Nominal p-value
rs3732253	rs2013162	0.87	0.25	0.12	0.14	0.03	0.19	0.06	0.81	0.74	0.47	0.45	0.91	0.44
rs1807968	rs2013162	0.83	0.59	0.5	0.81	0.52	0.39	0.24	0.32	0.34	0.77	0.74	0.93	0.87
rs374640	rs2013162	0.96	0.86	0.65	0.63	0.77	0.51	0.57	0.68	0.64	0.26	0.19	0.98	0.24
rs377122	rs2013162	0.44	0.16	0.12	0.45	0.45	0.94	0.79	0.04	0.03	0.64	0.98	0.09	0.11

SNPs		All Samples			Cleft Lip Only		Cleft Lip with or without Cleft Palate		Cleft Lip Only + Cleft Lip with or without Cleft Palate		Families with Mixed Cleft Types		Cleft Palate Only	
*TGFA*	*IRF6*	Initial FBAT p-value	Corrected p-value	Nominal p-value	Corrected p-value	Nominal p-value	Corrected p-value	Nominal p-value	Corrected p-value	Nominal p-value	Corrected p-value	Nominal p-value	Corrected p-value	Nominal p-value
rs3732253	rs2013162	0.87	0.25	0.12	0.9	0.82	0.75	0.55	0.31	0.21	0.18	0.23	0.28	0.85
rs1807968	rs2013162	0.83	0.59	0.5	0.003	0.01	0.7	0.59	0.61	0.35	0.06	0.86	0.35	0.24
rs374640	rs2013162	0.96	0.86	0.65	0.48	0.44	0.82	0.92	0.82	0.6	0.83	0.95	0.86	0.71
rs377122	rs2013162	0.44	0.16	0.12	0.72	0.39	0.05	0.05	0.88	0.98	0.82	0.81	0.19	0.85

** Cleft types, CL: cleft lip only; CLP: cleft lip with or without cleft palate; CLCLP: cleft lip only + cleft lip with or without cleft palate; MX: mixed cleft types; CP: cleft palate only.

*** Populations: Caucasians from USA and Europe.

We used genotypes for 154 cases from Latvia and genotype frequencies from the HapMap Project as a replication panel for this interaction between *TGFA* marker rs3732253 and *IRF6* rs2013162. The results also suggest *IRF6* and *TGFA* may interactively contribute to the risk for having an affected child (Table 8). The attributable fraction for these samples (AF = 0.04) suggests such interaction may account for ∼4% of the cases of cleft lip/palate in this particular population.

**Table 8:** Summary of the analysis with the Latvian cases (N = 154) with cleft lip/palate and 30 case-parent trios (90 individuals) from CEPH.

*IRF6*-*TGFA* Marker Genotypes*	Expected Frequency	Observed Frequency in Cleft Cases	p-value
CC-CC	0.2332	0.074074	0.002
CC-CT	0.176808	0.055556	0.005
CC-TT	0.013992	0.009259	0.5
AC-CC	0.26125	0.166667	0.1
AC-CT	0.198075	0.194444	0.02
AC-TT	0.015675	0.083333	0.03
AA-CC	0.0561	0.166667	0.005
AA-CT	0.042534	0.222222	0.0000001
AA-TT	0.003366	0.027778	0.27

* The *IRF6* marker used was rs2013162. The *TGFA* marker used was rs3732253.

### Results of Gene Expression Analysis

**Figure 1:**
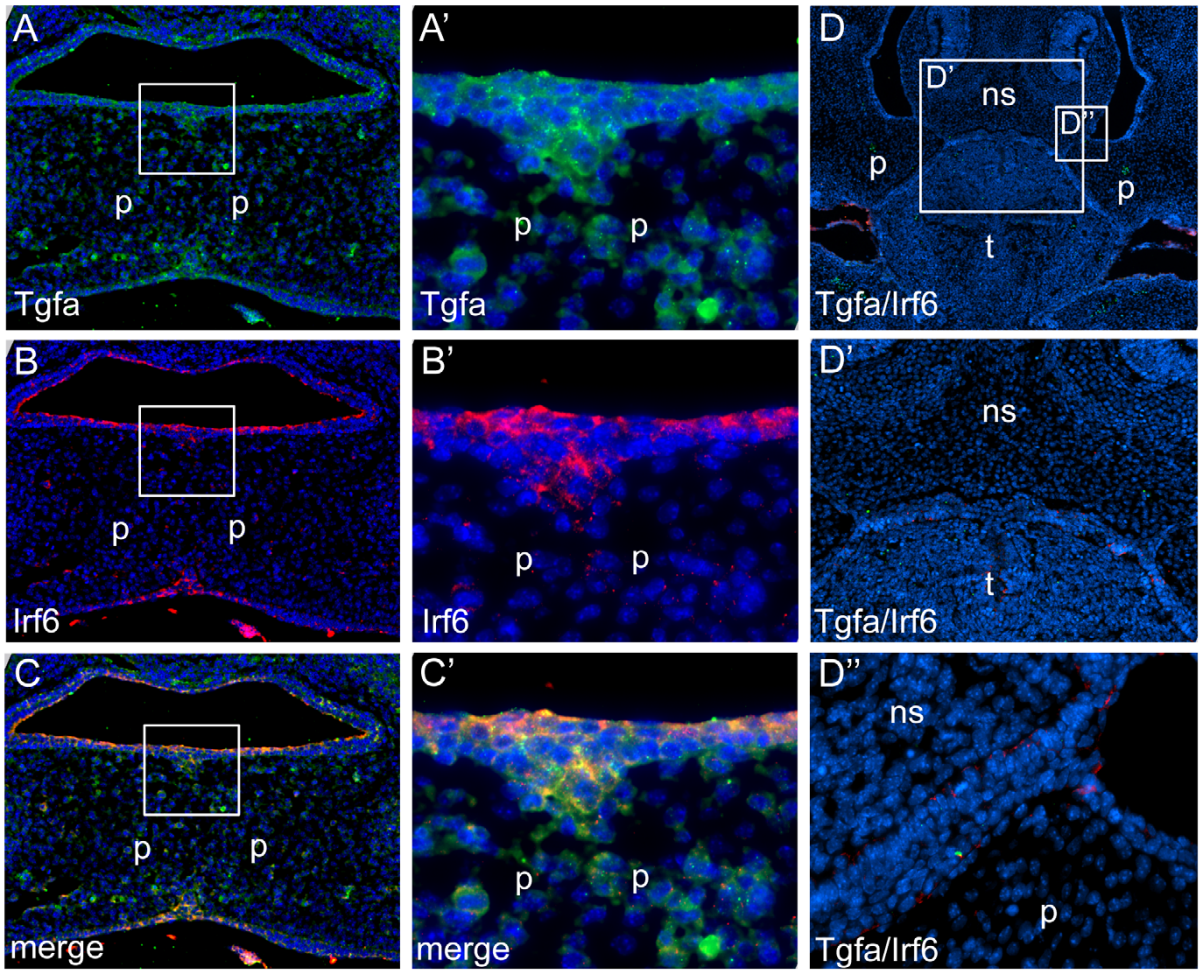
Loss of Tgfa expression in embryos that lack *Irf6*. Expression of Tgfa (A,A′), Irf6 (B, B′) and merge (C, C′) in coronal sections of E14.5 wild type murine embyos. Tgfa and Irf6 expression colocalized to oral and nasal epithelium and remaining medial edge epithelium. Magnification was 10× (A–C) and 40× (A′–C′) for boxed regions in panels A–C. No expression was observed for Tgfa and Irf6 in coronal sections of E14.5 embryos that lack *Irf6* (D). Regions of higher magnification are indicated (D′, D′′). Abbreviations are palate (p), tongue (t), nasal septum (ns).

**Figure 2:**
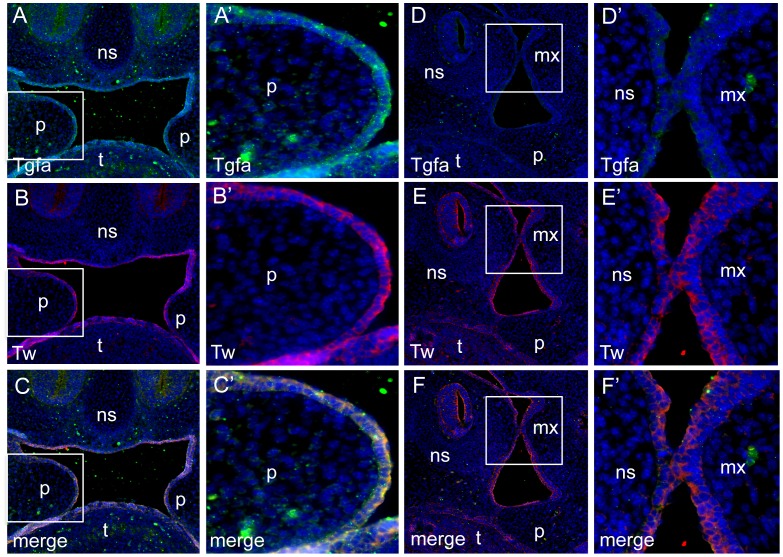
Loss of Tgfa, but not Twist, expression in embryos that lack *Irf6*. Expression of Tgfa (A,A′), Twist (B, B′) and merge (C, C′) in coronal sections of E13.5 wild type murine embyos. Tgfa and Twist expression colocalized to all oral epithelial surfaces of palate (p), tongue (t) and nasal septum (ns). Magnification was 10× (A–C) and 40× (A′–C′) for boxed regions in panels A–C. No expression was observed for Tgfa in coronal sections of E13.5 embryos that lack *Irf6* (D, F), but Twist expression was not affected (E, F). Regions of higher magnification are indicated (D′–F′)). Abbreviations are palate (p), tongue (t), nasal septum (ns).

We investigated localization of *Tgfa* and *Irf6* in wild type and *Irf6-*null mice at critical stages during palate development. We observed similar expression patterns for *Tgfa* and *Irf6* in wild-type mouse craniofacial tissues at embryonic days E13 through E15. Positive immunoreactivity was observed in both epithelial and mesenchymal tissues of the mouth and nasal cavities. Expression was also detected in the brain. At day E14.5, when palatal fusion takes place, both epithelial and mesenchymal cells in the palate were intensely stained for *Tgfa* and *Irf6* (Figure 1). In *Irf6* knockout mice, however, we did not see *Tgfa* expression at E14.5 (Figure 2).

## Discussion

Of all the genetic studies with cleft lip/palate, the association with *IRF6* is certainly the most consistent [6], and has been consistently replicated in multiple populations [8]–[22]. *IRF6* belongs to a gene family (IRFs) of transcription factors that regulate expression of interferons-α and –β after viral infections, and is a causal gene for Van der Woude’s syndrome which includes cleft lip/palate, pits on the lower lip, and tooth agenesis as part of the clinical phenotype [7]. Although the exact function of this gene remains unknown, polymorphisms in *IRF6* may account for ∼12% of all cleft cases in the background of other genes, with an association of a particular V274I allele with isolated cleft lip/palate among Filipinos [6].

Efforts are being made to unravel the specific role of *IRF6* in cleft lip/palate. Although direct sequencing of the coding regions of *IRF6* did not detect potential causative mutations, the causative variant(s) could be in linkage disequilibrium with V274I but reside in the regulatory element(s) of *IRF6* [32]. An additional possibility is that the V274I variant may be in linkage disequilibrium with a marker in another gene and such interaction might influence development of CL/P.

For many of the other genes previously associated with cleft lip/palate, including *TGFA*, a variety of positive and negative results have been reported, and *TGFA* has been largely ignored in the recent years. *TGFA* was the first gene associated to isolated cleft lip/palate in a case-control study [13] and was selected as a candidate gene because of its expression on palatal tissue in culture and its presence at high levels in the medial epithelial edge of the palatal shelves at the time of palatal fusion [42]. More recently, evidence of an excess of maternal transmission and possible interactions with maternal exposures to cigarette smoking, alcohol consumption and vitamin supplementation have been suggested to underlie the influence of *TGFA* in human clefting [43].

For this study, we used a collection of 1000 samples from cleft and control individuals from the Southeast region of Brazil [29]. Although the majority of residents of this region are descendants of Portuguese who migrated during the colonization years of Brazil, there is a substantial level of admixture confounding due to population stratification. To avoid biased results due to population stratification, the reported results reflect the analyses with individuals of self-reported Caucasian ethnicity only.

Markers located within and flanking the *IRF6* and *TGFA* genes were tested for association with cleft of the lip or palate under a case-control design. We found an association between a single nucleotide polymorphism in the intron of the *TGFA* gene with cleft lip/palate. There is no evidence suggesting this intronic variant is etiologic, however introns seem to affect virtually any step of mRNA maturation, including transcription initiation, transcription elongation, transcription termination, polydenylation, nuclear export, and mRNA stability [44]. Rs2902345 can potentially affect transcription, and is coincidently located flanking a region we suggested involved in segmental uniparental isodisomy in a case of maxillary lateral incisor and mandibular second premolar agenesis [45].

For *IRF6*, we found a positive association between the V274I polymorphism and complete left cleft lip/palate. In contrast to other studies [6], [8]–[22], all of which showed association of *IRF6* comparing only the three major cleft categories (cleft lip, cleft lip/palate and cleft palate), we only found positive association when comparing cleft subphenotypes with controls. Maybe *IRF6* is not a strong risk factor for clefting in Brazil, or maybe it has specific contributions, e.g. controlling the side of unilateral cleft. We also found an association between the V274I polymorphism with cases of cleft palate with impaction of permanent teeth. Tooth impaction occurs when, for some reason, the permanent teeth do not erupt and remain inside the alveolar bone. The reported prevalence in the general population is about 1 to 2.5%, and it has also been reported in children with clefts [46].

The frequency of the V274I polymorphism varies greatly depending on geographic origin [6]. For instance, rs642961 was found to be more useful in studies with populations of European origin, since V274I frequency in these groups is remarkably low [32]. But even this marker has distinct frequencies even populations originating from north Europe are compare with groups coming from the south (*i.e*., Hispanics versus non-Hispanics) [47]. Although rs642961 is located at a site suggested to be an *AP-2a* binding site promoter, we studied V274I in the Brazilians due to the expected allele frequency differences. Evidence however suggest that there is a contribution to cleft susceptibility at the *IRF6* locus but multiple genetic variants, rather than a single one, may have etiological roles in this defect [47], [48].

A common type of dental anomaly, tooth agenesis, has also been reported in association with this same *IRF6* variant by our group [28], [49]. Tooth agenesis is a common congenital anomaly where one or more permanent teeth are absent and a frequent observation in individuals with cleft lip/palate [29]. Previous evidence from tooth agenesis studies suggested *IRF6* and *TGFA* genes may interact [28]. Therefore, we hypothesized interaction between *IRF6* and *TGFA* may also be relevant to cleft lip/palate. We speculated on the attributable fraction for the interaction of *IRF6* and *TGFA* genes to the risk of cleft lip/palate using the Brazilian case-control sample and three additional family or case series data sets comprising a total of 8,717 individuals. We found statistical evidence of gene-gene interaction in all of these data sets and estimate such interaction could contribute from 1 percent to as much as 10 percent of cleft cases. These findings are in accordance with Zucchero et al. [6] who reported an attributable risk of cleft lip or palate of about 12 percent for *IRF6*. Those authors further stated the risk of recurrence is 9 percent among siblings in families with a history of cleft lip/palate where the child could have inherited the common risk allele. Taken together, these findings suggest individual *IRF6* status may be an important tool to revisit recurrence risk estimates for cleft lip/palate.

Our expression assays showed *Tgfa* and *Irf6* expression patterns are similar at critical stages for mouse palate development. However, *Tgfa* was not expressed in *Irf6* knock out mice, which suggests that *Tgfa* and *Irf6* may share common pathways and *Tgfa* may ultimately depend upon the *Irf6* expression status. Mice deficient for *Irf6* have abnormal skin, limb and craniofacial development, resultant from a primary defect in keratinocyte differentiation and proliferation. Furthermore, mice homozygous for the *Irf6* null allele have a cleft palate which seems to be caused by a defect in elevation, either as a primary defect or secondary to crowding of the craniofacial structures owing to the constrictive action of the skin or oral adhesions [40]. Deficiency of Tgfa has been shown to affect skin, hair and eye development although the presence of a cleft phenotype has not yet been described, and Tgfa has been regarded thus as a modifier gene [24]. Tgfa is expressed in a variety of developing and adult tissues and the majority of expression studies have assayed for the presence of mRNA, the levels of which may not correlate with the production and processing of the protein. Alternatively, there may be physiological redundancy among the ligands of the EGFR in some tissues. This hypothesis has gained some validity since the demonstration by bioinformatics approaches that the EGFR pathway contains regions of functional redundancy in its upstream parts that may alleviate the consequences of low EGF stimulus [50]. Although in theory these two genes have antagonizing functions - *Tgfa* as a growth factor capable of stimulating cellular proliferation and cellular differentiation, and *Irf6* as a regulator of keratinocyte proliferation and differentiation – our biological results further support the genetic interaction findings and warrant additional investigations.

The data presented here together with other evidence that suggest *p63* and *AP-2a* cooperate to regulate *IRF6* [32], [51]–[53] make us believe that a regulatory loop to coordinate epithelial proliferation and differentiation exist. Disruption of this loop by insufficient expression of *TGFA*, *p63*, *AP-2a* or any combination of these genes could lead to disruption in epithelial development. This disruption could lead to alterations such as clefts of the lip and palate and arresting of dental development, leading to tooth agenesis. Since TGFA, IRF6, and p63 are known to be involved in cancer [24], [54], [55], and in the view of our recent findings that cleft lip and palate families report more cancer [56]–[60], these gene-gene interactions might not only explain susceptibility to oral clefts, but also cancer.

Cleft lip/palate is a complex and heterogeneous disorder and a likely scenario is that variation in more than one gene underlies the isolated cleft lip/palate [61]. Additional studies should be realized regarding *IRF6-TGFA* interaction in other populations and if confirmed, these results could be used to revisit estimates of the recurrence rates of clefting.

## Materials and Methods

### Subjects

The subjects of this study, their cleft subphenotypes and characteristics of dental anomalies have been previously described in detail [29]. Written informed consent was obtained from all participants in the study. Parents or legal guardians provided written consent on behalf of the minors/children participants involved in the study. The sample consisted of 500 individuals with clefts in treatment at the Hospital of Rehabilitation and Craniofacial Anomalies of the University of São Paulo, Bauru, Brazil. Of these, 400 had cleft lip with cleft palate (168 with left cleft lip, 154 with bilateral cleft lip, 76 with right cleft lip, and 2 median clefts), six had cleft lip only (two on the right side and four on the left side), 66 had cleft palate only and 28 had unknown cleft types. The control group comprised 500 healthy, non-related individuals with no history of syndromic clefting, whom were mostly patients and students at Bauru Dental School.

We detected evidence of confounding due to population stratification within this Brazilian sample and therefore, for this study, we have included only the individuals of Caucasian ethnicity (hereby defined as Brazilians of Caucasian descent to the third generation and without any African or Japanese descent). Hence, 406 individuals with clefts and 285 control individuals were included in the current analysis (Table 1).

The study was conducted with the consent of the participants and approved by the Research and Ethics Committee of the University of São Paulo, Bauru and University of Pittsburgh. In the case of children under 15 years of age, authorization was also requested from their parents or from their legal guardian. Buccal epithelial cells were collected from each individual as source of genomic DNA. Procedures for buccal cell collection and DNA extraction were performed as described elsewhere [30], [31].

### Genotyping

Genotyping was performed using Taqman or SYBR® Green chemistries (Applied Biosystems) on an automatic sequence-detection instrument (ABI Prism 7900HT, Applied Biosystems). Reactions were carried out with the use of standard conditions as suggested by the manufacturer.

SNP selection was based on previous reports. Six markers were genotyped in/nearby the *IRF6* gene [6], [32]. In addition, six intragenic markers were used for *TGFA* [23]. Details of the markers are presented in Table 2. For the C3296T (rs2166975) and C3827T (rs1058213) variants in *TGFA*, we used allele-specific primers according to previously published protocols [33]. The primer sequences were: for C3296T, forward 5′CTTATTTTCCCAACGTGGCC 3′; reverse (for C allele), 5′CTCCTCTGGGCTCTTCTG 3′, reverse (for T allele), 5′TCCTCCTCTGGGCTCTTCTA 3′; for C3827T, forward 5′CTTATTTTCCCAACGTGGCC 3′; reverse (for C allele) 5′CTCCTCTGGGCTCTTCTG 3′, reverse (for T allele) 5′TCCTCCTCTGGGCTCTTCTA 3′. PCR conditions were the same for both variants: 95°C for 10 min (1 cycle), 95°C for 15 sec, and 56°C for 40 sec (55 cycles).

Since some of these markers had not yet been previously genotyped in Brazilians, we calculated linkage disequilibrium between all markers using the Graphical Overview of Linkage Disequilibrium (GOLD) software using both the squared correlation coefficient (r^2^, above the diagonal) and Lewontin’s standardized disequilibrium coefficient (D′, below the diagonal) [34] (Table 3).

All SNP markers were first tested on a collection of samples from the Centre d’Étude du Polymorphisme Humain (CEPH).

### Statistical Analysis

#### Hardy-Weinberg equilibrium and case-control analyses

Chi-square statistics were used to assess adherence to Hardy-Weinberg equilibrium between cases and controls. There was no evidence of deviation from Hardy-Weinberg equilibrium for both groups (data not shown).

For case-control comparisons in the Brazilian samples, Chi-square test was used to assess association of markers with each cleft subphenotype. Bonferroni correction was applied considering the number of variables and tests performed, and *P*-values below 0.0002 (12 SNPs, and 17 phenotypes: 0.05/204) were considered significant.

#### IRF6-TGFA interaction and attributable fraction

The *IRF6* and *TGFA* markers yielding the most significant associations were used to infer the overall contribution of their interaction to nonsyndromic cleft lip/palate in Brazilian cases and controls. We calculated the attributable fraction (AF) for the associated *IRF6* and *TGFA* alleles as the proportion of cleft cases in a population that could be attributed to the interaction terms, assuming true causality. We calculated AF =  f (R-1)/R where f is the frequency of the risk factor in the population and R is the measure of relative risk (35). To obtain f, we used the number of cases presenting at least one copy of overrepresented alleles compared to controls for both markers and divided by the total number of cases. We used the relative risk values for heritability of clefting in Brazilians in the State of São Paulo (RR = 4.96) as reported by Lofredo et al. [36].

We also studied three distinct data sets to replicate evidence of interaction between *IRF6* and *TGFA*. Genotypes available for an additional 142 case-parent trios from ECLAMC (Latin American Collaborative Study of Congenital Malformations) were also included in the analysis for interaction between the *IRF6* V274I variant (rs2235371) and *TGFA* C3827T (rs1058213) by observing transmission of the associated alleles at each gene from parents heterozygous for both of the markers using the parental haplotypes not present in the affected child as controls [37]. ECLAMC is a hospital-based birth defects registry study that included sites in Argentina, Bolivia, Brazil, Chile, Ecuador, Paraguay, Uruguay and Venezuela. Genotypes and alleles at each *IRF6* and *TGFA* marker were tested for association with cleft lip/palate using of Family-Based Association Test (FBAT) [38] software in the ECLAMC cohort. The study was conducted with the consent of the participants and approved by the Research and Ethics Committee of the CEMIC (Centro de Educación Médica e Investigaciones Clínicas “Norberto Quirno”), Buenos Aires, Argentina and University of Pittsburgh. Written informed consent was obtained from all participants in the study. Parents or legal guardians provided written consent on behalf of the minors/children participants involved in the study.

In addition, data from a population pool consisting of 7,047 people from family studies of CL/P sampled from Colombia, USA, India, Spain, Philippines, China and Turkey were also analyzed. Details regarding these are provided elsewhere [17]. In brief, most of these families were extended multiplex kindreds, i.e. multigenerational families with two or more affected individuals. The phenotype was CL/P, i.e. for families to be included, it was necessary that the proband have CL/P (i.e. no other anomalies) and that no other family member have an indication of an orofacial syndrome (e.g. lip pits). Each study population included evaluations of family members by clinical geneticists to rule out syndromic forms of CL/P. For this analysis, we considered the per-family Z-scores for each SNP from FBAT and performed correlation and logistic regression between each *IRF6* and *TGFA* SNPs to generate interaction p-values. We used the odds ratio of the associated alleles as a measure of the relative risk in this pooled population. The study was conducted with the consent of the participants and approved by the Research and Ethics Committee of the University of Pittsburgh. Written informed consent was obtained from all participants in the study. Parents or legal guardians provided written consent on behalf of the minors/children participants involved in the study.

As a replication panel for these interaction analyses, we used genotypes of 154 cases with isolated cleft lip/palate from Latvia to test for interaction between markers in the *IRF6* and *TGFA* genes. Since no Latvian control samples were available, comparison data from this analysis was drawn from genotype frequency in 30 U.S. trios (mother/father/offspring), which were collected in 1980 from U.S. residents with northern and western European ancestry by the Centre d’Etude du Polymorphisme Humain (CEPH) available at the HapMap Project (http://hapmap.ncbi.nlm.nih.gov/). Although these control samples are presumed to be of Northern Europe origin that is the potential that population substructures are not matched in this set of cases and controls. We did the same calculations used for the Brazilian and ECLAMC datasets. To obtain f, we used the frequency of the over-represented haplotype among the CEPH controls. We used a relative risk value of 1.0 in the Latvians due to the very high frequency of maternal smoking in the population (67% to 81%) as reported by Patla et al. [39]. Maternal smoking is known to increase the susceptibility to clefts approximately 1.5 times [61] and in a population that have a frequency of smokers as high as the Latvians, we decided that would be appropriate to not input relative risk values higher than 1.0 for this calculation. The study was conducted with the consent of the participants and approved by the Research and Ethics Committee of the Riga Stradins University and University of Pittsburgh. Written informed consent was obtained from all participants in the study. Parents or legal guardians provided written consent on behalf of the minors/children participants involved in the study.

Overall, 8,717 individuals were used in this test of interaction between *IRF6* and *TGFA*.

### Fluorescent Immunostaining

Expression of Tgfa and Irf6 proteins was performed on paraffin sections from heads of wild type and Irf6 null embryos at E13.5 and 14.5. Maintenance and handling of mice were approved by the Animal Care Unit at Michigan State University. Tissues were deparaffinized and rehydrated in a series of ethanol dilutions. Slides were boiled for 5 min in 0.08% saponin in BPS for antigen retrieval. Sections were blocked with 10% normal goat serum in 1% PBS-BSA for 1 hr, then incubated overnight at 4 šC with the following primary antibodies: monoclonal mouse anti-Tgfa (1∶150, clone 213-4.4, GeneTex, Irvine, CA) and polyclonal rabbit anti-Irf6 (1∶500, Irf6-SPEA). After rinsing in PBS, sections were incubated with secondary antibodies conjugated to Alexa Fluorophore 488 or 555 (Molecular Probes, Invitrogen, CA). The nuclei were counterstained with DAPI in PBS (1∶1000). The images were taken using a Nikon Eclipse 90i fluorescent microscope.

To investigate if *Tgfa* was influenced by *Irf6,* we also investigated *Tgfa* expression in the *Irf6* knockout mice generated by Ingraham et al. [40] using the methods and reagents as described above.

Skin tissue sections were used as positive controls for *Tgfa* [41] and *Irf6* [40]. To confirm the specificity of the immunostaining, primary antibodies were substituted with PBS (for *Tgfa*) and normal rabbit serum (for *Irf6*). No immunoreactivity was detected in any of the negative control sections.

## Supporting Information

Table S1 Genotyping data of ECLAMC cleft lip and palate trios.
(PDF)
