# Supplementary material for: Interaction between IRF6 and TGFA Genes Contribute to the Risk of Nonsyndromic Cleft Lip/Palate
Source: PLoS One. 2012 Sep 20;7(9):e45441. doi: 10.1371/journal.pone.0045441 (PMC3447924; doi:10.1371/journal.pone.0045441)
Supplement: Table S1 — Genotyping data of ECLAMC cleft lip and palate trios. (PDF) [file pone.0045441.s001.pdf]

Table S1. Raw data used to generate attributable fraction results for ECLAMC population.
We used Haplotype Risk Calculations [37] using parental haplotypes
not present in the affected child as control sample.

						Cases		Controls	
						IRF6	TGFA	IRF6	TGFA
Family ID	Ind ID	Founder 1	Founder 2	Gender	Affection	rs2235371	rs1058213	rs2235371	rs1058213
1	1	2	3	2	2	CC	CC	CT	CC
1	2	0	0	2	1	CT	CC		
1	3	0	0	1	1	CC	CC		
2	1	2	3	2	2	CC	CC	CT	CC
2	2	0	0	2	1	CT	CC		
2	3	0	0	1	1	CC	CC		
3	1	2	3	1	2	CC	CC	CT	CC
3	2	0	0	2	1	CC	CC		
3	3	0	0	1	1	CT	CC		
6	1	2	3	1	2	CC	CC	CC	CC
6	2	0	0	1	1	CC	CC		
6	3	0	0	2	1	CC	CC		
12	1	2	3	1	2	CT	CC	CT	CC
12	2	0	0	1	1	TT	CC		
12	3	0	0	2	1	CC	CC		
13	1	2	3	1	2	CC	CC	CC	CT
13	2	0	0	1	1	CC	CT		
13	3	0	0	2	1	CC	CC		
15	1	2	3	1	2	CC	CC	CC	CC
15	2	0	0	1	1	CC	CC		
15	3	0	0	2	1	CC	CC		
17	1	2	3	2	2	CC	CC	CC	CC
17	2	0	0	1	1	CC	CC		
17	3	0	0	2	1	CC	CC		
18	1	2	3	2	2	CC	CC	CT	CT
18	2	0	0	1	1	CT	CC		
18	3	0	0	2	1	CC	CT		
20	1	2	3	2	2	CC	CC	CC	CC
20	2	0	0	2	1	CC	CC		
20	3	0	0	1	1	CC	CC		
21	1	2	3	1	2	CC	CC	CC	CC
21	2	0	0	2	1	CC	CC		
21	3	0	0	1	1	CC	CC		
23	1	2	3	1	2	CC	CC	CC	CT
23	2	0	0	2	1	CC	CC		
23	3	0	0	1	1	CC	CT		
25	1	2	3	2	2	CC	CT	CC	CT
25	2	0	0	2	1	CC	CC		
25	3	0	0	1	1	CC	TT		
26	1	2	3	2	2	CT	CC	CC	CC
26	2	0	0	2	1	CT	CC		
26	3	0	0	1	1	CC	CC		
30	1	2	3	1	2	CT	CC	TT	CC
30	2	0	0	2	1	TT	CC		
30	3	0	0	1	1	CT	CC		
31	1	2	3	1	2	CC	CC	CC	CC
31	2	0	0	2	1	CC	CC		
31	3	0	0	1	1	CC	CC		
32	1	2	3	2	2	CT	CT	CT	CC
32	2	0	0	2	1	TT	CC		
32	3	0	0	1	1	CC	CT		
34	1	2	3	1	2	CC	CC	CC	CT
34	2	0	0	2	1	CC	CT		
34	3	0	0	1	1	CC	CC		
35	1	2	3	1	2	CC	CT	CT	CC
35	2	0	0	2	1	CT	CC		
35	3	0	0	1	1	CC	CT		
36	1	2	3	2	2	CT	CC	CT	CT
36	2	0	0	2	1	TT	CC		
36	3	0	0	1	1	CC	CT		
38	1	2	3	2	2	CC	CC	CC	CC
38	2	0	0	2	1	CC	CC		
38	3	0	0	1	1	CC	CC		
40	1	2	3		2	CC	TT	CC	CC
40	2	0	0	2	1	CC	CT		
40	3	0	0	1	1	CC	CT		
41	1	2	3	1	2	CC	CC	CC	CC
41	2	0	0	2	1	CC	CC		
41	3	0	0	1	1	CC	CC		
45	1	2	3	1	2	CT	CC	CT	CC
45	2	0	0	2	1	CT	CC		
45	3	0	0	1	1	CT	CC		
46	1	2	3	2	2	CC	CC	CC	CT
46	2	0	0	2	1	CC	CC		
46	3	0	0	1	1	CC	CT		
48	1	2	3		2	CT	CC	CC	CC
48	2	0	0	2	1	CC	CC		
48	3	0	0	1	1	CT	CC		
49	1	2	3		2	CC	CT	CT	CC
49	2	0	0	2	1	CC	CT		
49	3	0	0	1	1	CT	CC		
50	1	2	3		2	CC	CC	CC	CT

50	2	0	0	2	1	CC	CC		
50	3	0	0	1	1	CC	CT		
51	1	2	3	1	2	CT	CT	CT	CC
51	2	0	0	2	1	CT	CT		
51	3	0	0	1	1	CT	CC		
52	1	2	3		2	CC	CC	CT	CC
52	2	0	0	2	1	CT	CC		
52	3	0	0	1	1	CC	CC		
53	1	2	3	1	2	CC	CC	CC	CT
53	2	0	0	2	1	CC	CC		
53	3	0	0	1	1	CC	CT		
55	1	2	3	1	2	CC	CT	CC	CT
55	2	0	0	2	1	CC	CC		
55	3	0	0	1	1	CC	TT		
56	1	2	3	1	2	CC	CC	CT	CT
56	2	0	0	2	1	CT	CT		
56	3	0	0	1	1	CC	CC		
57	1	2	3	1	2	CT	CC	CT	CT
57	2	0	0	2	1	CC	CT		
57	3	0	0	1	1	TT	CC		
59	1	2	3	1	2	CT	CC	CC	CC
59	2	0	0	2	1	CC	CC		
59	3	0	0	1	1	CT	CC		
60	1	2	3	1	2	CC	CC	CT	CC
60	2	0	0	2	1	CT	CC		
60	3	0	0	1	1	CC	CC		
61	1	2	3	1	2	CC	CC	CT	CC
61	2	0	0	2	1	CT	CC		
61	3	0	0	1	1	CC	CC		
62	1	2	3	1	2	CC	TT	CT	CT
62	2	0	0	2	1	CC	CT		
62	3	0	0	1	1	CT	TT		
63	1	2	3	1	2	CC	CC	TT	CC
63	2	0	0	2	1	CT	CC		
63	3	0	0	1	1	CT	CC		
64	1	2	3	1	2	CC	CC	CC	CT
64	2	0	0	2	1	CC	CC		
64	3	0	0	1	1	CC	CT		
66	1	2	3	2	2	CC	CT	CT	CC
66	2	0	0	2	1	CT	CT		
66	3	0	0	1	1	CC	CC		
73	1	2	3	1	2	CC	CT	TT	CC
73	2	0	0	2	1	CT	CT		
73	3	0	0	1	1	CT	CC		
76	1	2	3	1	2	CC	CC	CC	CC
76	2	0	0	2	1	CC	CC		
76	3	0	0	1	1	CC	CC		
77	1	2	3	1	2	TT	CC	CT	CC
77	2	0	0	2	1	TT	CC		
77	3	0	0	1	1	CT	CC		
78	1	2	3	1	2	CC	CC	CC	CT
78	2	0	0	2	1	CC	CT		
78	3	0	0	1	1	CC	CC		
79	1	2	3	1	2	CC	CC	CC	CC
79	2	0	0	2	1	CC	CC		
79	3	0	0	1	1	CC	CC		
81	1	2	3	1	2	CC	CC	CC	CT
81	2	0	0	2	1	CC	CT		
81	3	0	0	1	1	CC	CC		
82	1	2	3	1	2	CC	CC	CT	CC
82	2	0	0	2	1	CC	CC		
82	3	0	0	1	1	CT	CC		
83	1	2	3	1	2	CT	CC	CT	TT
83	2	0	0	2	1	CT	CT		
83	3	0	0	1	1	CT	CT		
85	1	2	3	2	2	CC	CC	CT	CC
85	2	0	0	2	1	CC	CC		
85	3	0	0	1	1	CT	CC		
87	1	2	3	1	2	CC	CC	CC	CC
87	2	0	0	2	1	CC	CC		
87	3	0	0	1	1	CC	CC		
88	1	2	3	1	2	CC	CC	CC	CC
88	2	0	0	2	1	CC	CC		
88	3	0	0	1	1	CC	CC		
89	1	2	3	1	2	CC	CC	CC	CC
89	2	0	0	2	1	CC	CC		
89	3	0	0	1	1	CC	CC		
90	1	2	3	1	2	CC	CC	CT	CC
90	2	0	0	2	1	CT	CC		
90	3	0	0	1	1	CC	CC		
93	1	2	3	1	2	CC	CC	TT	CC
93	2	0	0	2	1	CT	CC		
93	3	0	0	1	1	CT	CC		
94	1	2	3	1	2	CT	CC	CT	CC
94	2	0	0	2	1	CT	CC		
94	3	0	0	1	1	CT	CC		
95	1	2	3	1	2	CC	CC	CC	CC
95	2	0	0	2	1	CC	CC		

95	3	0	0	1	1	CC	CC		
96	1	2	3	1	2	CT	CC	CC	CC
96	2	0	0	2	1	CC	CC		
96	3	0	0	1	1	CT	CC		
97	1	2	3	1	2	CT	CT	CC	CT
97	2	0	0	2	1	CC	CT		
97	3	0	0	1	1	CT	CT		
98	1	2	3	1	2	CT	CC	CC	CC
98	2	0	0	2	1	CC	CC		
98	3	0	0	1	1	CT	CC		
99	1	2	3	2	2	CT	CC	CC	CC
99	2	0	0	2	1	CT	CC		
99	3	0	0	1	1	CC	CC		
100	1	2	3	2	2	CC	CC	CC	CC
100	2	0	0	2	1	CC	CC		
100	3	0	0	1	1	CC	CC		
101	1	2	3	1	2	CC	CC	CC	CC
101	2	0	0	2	1	CC	CC		
101	3	0	0	1	1	CC	CC		
102	1	2	3	1	2	CC	CC	CC	CC
102	2	0	0	2	1	CC	CC		
102	3	0	0	1	1	CC	CC		
103	1	2	3	2	2	CC	CC	CC	CC
103	2	0	0	2	1	CC	CC		
103	3	0	0	1	1	CC	CC		
104	1	2	3	1	2	CC	CC	CC	CT
104	2	0	0	2	1	CC	CT		
104	3	0	0	1	1	CC	CC		
105	1	2	3	1	2	CC	CC	CC	CT
105	2	0	0	2	1	CC	CC		
105	3	0	0	1	1	CC	CT		
106	1	2	3	1	2	CC	CC	CT	CC
106	2	0	0	2	1	CT	CC		
106	3	0	0	1	1	CC	CC		
107	1	2	3	1	2	CT	CC	CT	CT
107	2	0	0	2	1	CT	CC		
107	3	0	0	1	1	CT	CT		
108	1	2	3	2	2	CC	CC	CC	CC
108	2	0	0	2	1	CC	CC		
108	3	0	0	1	1	CC	CC		
109	1	2	3	2	2	CC	CT	CT	CC
109	2	0	0	2	1	CC	CC		
109	3	0	0	1	1	CT	CT		
110	1	2	3	1	2	CC	CC	TT	CC
110	2	0	0	2	1	CT	CC		
110	3	0	0	1	1	CT	CC		
111	1	2	3	1	2	CT	CT	CC	CC
111	2	0	0	2	1	CT	CT		
111	3	0	0	1	1	CC	CC		
112	1	2	3	1	2	CT	CC	CT	CC
112	2	0	0	2	1	CT	CC		
112	3	0	0	1	1	CT	CC		
113	1	2	3	1	2	CC	CT	CC	CT
113	2	0	0	2	1	CC	TT		
113	3	0	0	1	1	CC	CC		
116	1	2	3	1	2	CC	CT	CC	CC
116	2	0	0	2	1	CC	CT		
116	3	0	0	1	1	CC	CC		
118	1	2	3	1	2	CC	CC	TT	CC
118	2	0	0	2	1	CT	CC		
118	3	0	0	1	1	CT	CC		
119	1	2	3	1	2	CT	CC	CT	CC
119	2	0	0	2	1	CT	CC		
119	3	0	0	1	1	CT	CC		
120	1	2	3	1	2	CT	CC	CT	CC
120	2	0	0	2	1	CC	CC		
120	3	0	0	1	1	TT	CC		
121	1	2	3	2	2	CC	CC	CT	CT
121	2	0	0	2	1	CC	CC		
121	3	0	0	1	1	CT	CT		
122	1	2	3	1	2	CC	CC	CC	CC
122	2	0	0	2	1	CC	CC		
122	3	0	0	1	1	CC	CC		
123	1	2	3	2	2	CT	CC	TT	CC
123	2	0	0	2	1	CT	CC		
123	3	0	0	1	1	TT	CC		
124	1	2	3	2	2	CT	CC	CC	CC
124	2	0	0	2	1	CT	CC		
124	3	0	0	1	1	CC	CC		
126	1	2	3	1	2	CC	CC	TT	CT
126	2	0	0	2	1	CT	CC		
126	3	0	0	1	1	CT	CT		
127	1	2	3	1	2	CC	CC	CT	CT
127	2	0	0	2	1	CC	CT		
127	3	0	0	1	1	CT	CC		
128	1	2	3	1	2	CC	CC	CT	CC
128	2	0	0	2	1	CC	CC		
128	3	0	0	1	1	CT	CC		

129	1	2	3	1	2	CT	CC	CT	CC
129	2	0	0	2	1	TT	CC		
129	3	0	0	1	1	CC	CC		
130	1	2	3	2	2	CC	CC	CC	CC
130	2	0	0	2	1	CC	CC		
130	3	0	0	1	1	CC	CC		
131	1	2	3		2	CC	CC	CC	CC
131	2	0	0	2	1	CC	CC		
131	3	0	0	1	1	CC	CC		
134	1	2	3		2	CC	CC	CC	CC
134	2	0	0	2	1	CC	CC		
134	3	0	0	1	1	CC	CC		
137	1	2	3		2	CT	CC	TT	CC
137	2	0	0	2	1	CT	CC		
137	3	0	0	1	1	TT	CC		
143	1	2	3	2	2	CT	CT	CT	CC
143	2	0	0	2	1	CT	CC		
143	3	0	0	1	1	CT	CT		
144	1	2	3	2	2	CC	CC	CC	CC
144	2	0	0	2	1	CC	CC		
144	3	0	0	1	1	CC	CC		
145	1	2	3	1	2	CC	CT	CT	CC
145	2	0	0	2	1	CT	CC		
145	3	0	0	1	1	CC	CC		
146	1	2	3	1	2	CT	CC	CT	CC
146	2	0	0	2	1	TT	CC		
146	3	0	0	1	1	CC	CC		
157	1	2	3	1	2	CC	CT	CT	CT
157	2	0	0	2	1	CT	CT		
157	3	0	0	1	1	CC	CT		
158	1	2	3	1	2	CT	CC	CT	CC
158	2	0	0	2	1	CT	CC		
158	3	0	0	1	1	CT	CC		
163	1	2	3	1	2	CC	CC	CT	TT
163	2	0	0	2	1	CC	CT		
163	3	0	0	1	1	CT	CT		
164	1	2	3	1	2	CC	CC	CC	CC
164	2	0	0	2	1	CC	CC		
164	3	0	0	1	1	CC	CC		
167	1	2	3		2	CC	CC	CC	CT
167	2	0	0	2	1	CC	CT		
167	3	0	0	1	1	CC	CC		
170	1	2	3		2	CC	CC	CC	CC
170	2	0	0	2	1	CC	CC		
170	3	0	0	1	1	CC	CC		
172	1	2	3		2	CT	CC	TT	CC
172	2	0	0	2	1	CT	CC		
172	3	0	0	1	1	TT	CC		
173	1	2	3		2	CC	CC	CC	CC
173	2	0	0	2	1	CC	CC		
173	3	0	0	1	1	CC	CC		
174	1	2	3		2	CC	CT	TT	CC
174	2	0	0	2	1	CT	CT		
174	3	0	0	1	1	CT	CC		
175	1	2	3		2	CC	CT	CC	CC
175	2	0	0	2	1	CC	CC		
175	3	0	0	1	1	CC	CT		
176	1	2	3		2	CC	CC	CT	CT
176	2	0	0	2	1	CC	CT		
176	3	0	0	1	1	CT	CC		
177	1	2	3	1	2	CT	CC	CC	CC
177	2	0	0	2	1	CT	CC		
177	3	0	0	1	1	CC	CC		
178	1	2	3	2	2	CC	CC	CT	CC
178	2	0	0	2	1	CT	CC		
178	3	0	0	1	1	CC	CC		
179	1	2	3	2	2	CC	CT	CC	CT
179	2	0	0	2	1	CC	CT		
179	3	0	0	1	1	CC	CT		
180	1	2	3	2	2	CT	CC	CC	CC
180	2	0	0	2	1	CC	CC		
180	3	0	0	1	1	CT	CC		
181	1	2	3	1	2	CC	CT	CT	CC
181	2	0	0	2	1	CC	CT		
181	3	0	0	1	1	CT	CC		
182	1	2	3	1	2	CT	CC	CC	CC
182	2	0	0	2	1	CT	CC		
182	3	0	0	1	1	CC	CC		
183	1	2	3	1	2	CT	CT	CT	CC
183	2	0	0	2	1	CT	CT		
183	3	0	0	1	1	CT	CC		
185	1	2	3	2	2	CC	CC	CC	CC
185	2	0	0	2	1	CC	CC		
185	3	0	0	1	1	CC	CC		
186	1	2	3	1	2	CT	CC	CC	CC
186	2	0	0	2	1	CC	CC		
186	3	0	0	1	1	CT	CC		
188	1	2	3	2	2	CT	CC	CC	CC

188	2	0	0	2	1	CT	CC		
188	3	0	0	1	1	CC	CC		
189	1	2	3	2	2	CC	CC	CC	TT
189	2	0	0	2	1	CC	CT		
189	3	0	0	1	1	CC	CT		
191	1	2	3	1	2	CT	CC	CC	CT
191	2	0	0	2	1	CC	CC		
191	3	0	0	1	1	CT	CT		
192	1	2	3	2	2	CC	CC	CT	CC
192	2	0	0	2	1	CC	CC		
192	3	0	0	1	1	CT	CC		
193	1	2	3	1	2	CT	CC	CT	CC
193	2	0	0	2	1	TT	CC		
193	3	0	0	1	1	CC	CC		
195	1	2	3		2	CC	CC	CT	CC
195	2	0	0	2	1	CT	CC		
195	3	0	0	1	1	CC	CC		
196	1	2	3		2	CT	CC	CT	CC
196	2	0	0	2	1	CC	CC		
196	3	0	0	1	1	TT	CC		
197	1	2	3		2	CT	CC	CT	CC
197	2	0	0	2	1	CC	CC		
197	3	0	0	1	1	TT	CC		
198	1	2	3		2	CT	CC	CC	CC
198	2	0	0	2	1	CT	CC		
198	3	0	0	1	1	CC	CC		
199	1	2	3		2	CC	CC	CC	CC
199	2	0	0	2	1	CC	CC		
199	3	0	0	1	1	CC	CC		
200	1	2	3		2	CC	CC	TT	CC
200	2	0	0	2	1	CT	CC		
200	3	0	0	1	1	CT	CC		
201	1	2	3		2	CT	CC	CC	CC
201	2	0	0	2	1	CC	CC		
201	3	0	0	1	1	CT	CC		
202	1	2	3		2	CC	CC	TT	CC
202	2	0	0	2	1	CT	CC		
202	3	0	0	1	1	CT	CC		
206	1	2	3		2	CC	CC	CT	CT
206	2	0	0	2	1	CC	CT		
206	3	0	0	1	1	CT	CC		
207	1	2	3		2	CT	CC	CT	CC
207	2	0	0	2	1	CT	CC		
207	3	0	0	1	1	CT	CC		
208	1	2	3		2	CT	CT	CT	CC
208	2	0	0	2	1	CT	CC		
208	3	0	0	1	1	CT	CT		
209	1	2	3		2	CC	CC	CC	CC
209	2	0	0	2	1	CC	CC		
209	3	0	0	1	1	CC	CC		
210	1	2	3		2	CT	CC	CT	CC
210	2	0	0	2	1	CT	CC		
210	3	0	0	1	1	CT	CC		
211	1	2	3		2	CC	CC	TT	CT
211	2	0	0	2	1	CT	CT		
211	3	0	0	1	1	CT	CC		
212	1	2	3		2	CC	CC	TT	CC
212	2	0	0	2	1	CT	CC		
212	3	0	0	1	1	CT	CC		
213	1	2	3	1	2	CC	CT	CC	CC
213	2	0	0	2	1	CC	CT		
213	3	0	0	1	1	CC	CC		
214	1	2	3	1	2	CC	CC	CT	CC
214	2	0	0	2	1	CT	CC		
214	3	0	0	1	1	CC	CC		
215	1	2	3	2	2	CC	CC	CC	CC
215	2	0	0	2	1	CC	CC		
215	3	0	0	1	1	CC	CC		
216	1	2	3	1	2	CC	CC	CC	CT
216	2	0	0	2	1	CC	CC		
216	3	0	0	1	1	CC	CT		
217	1	2	3	2	2	CC	CC	CC	CC
217	2	0	0	2	1	CC	CC		
217	3	0	0	1	1	CC	CC		
218	1	2	3	2	2	CC	CT	CC	CT
218	2	0	0	2	1	CC	CT		
218	3	0	0	1	1	CC	CT		
219	1	2	3	2	2	CT	CC	CT	CC
219	2	0	0	2	1	CT	CC		
219	3	0	0	1	1	CT	CC		
222	1	2	3	1	2	CT	TT	CT	CT
222	2	0	0	2	1	CC	TT		
222	3	0	0	1	1	TT	CT		
223	1	2	3	1	2	CC	CC	CC	CT
223	2	0	0	2	1	CC	CC		
223	3	0	0	1	1	CC	CT		
224	1	2	3	2	2	CC	CC	CC	CT
224	2	0	0	2	1	CC	CT		

224	3	0	0	1	1	CC	CC		
225	1	2	3	2	2	CC	CC	CC	CC
225	2	0	0	2	1	CC	CC		
225	3	0	0	1	1	CC	CC		
226	1	2	3		2	CT	CC	CT	CC
226	2	0	0	2	1	CT	CC		
226	3	0	0	1	1	CT	CC		
228	1	2	3	2	2	CC	CC	CT	TT
228	2	0	0	2	1	CC	CT		
228	3	0	0	1	1	CT	CT		
230	1	2	3	1	2	CT	CC	CC	CC
230	2	0	0	2	1	CT	CC		
230	3	0	0	1	1	CC	CC		
235	1	2	3		2	CC	CC	CT	CC
235	2	0	0	2	1	CT	CC		
235	3	0	0	1	1	CC	CC		
236	1	2	3		2	CC	CC	CC	CC
236	2	0	0	2	1	CC	CC		
236	3	0	0	1	1	CC	CC		
237	1	2	3		2	CC	CC	CC	CT
237	2	0	0	2	1	CC	CT		
237	3	0	0	1	1	CC	CC		
238	1	2	3		2	TT	CC	CT	CC
238	2	0	0	2	1	CT	CC		
238	3	0	0	1	1	TT	CC		
239	1	2	3		2	CC	CC	CC	CC
239	2	0	0	2	1	CC	CC		
239	3	0	0	1	1	CC	CC		
241	1	2	3	2	2	CC	CC	CC	CC
241	2	0	0	2	1	CC	CC		
241	3	0	0	1	1	CC	CC		
242	1	2	3	2	2	CC	CC	CT	CC
242	2	0	0	2	1	CT	CC		
242	3	0	0	1	1	CC	CC		
244	1	2	3	2	2	CC	CC	CC	CC
244	2	0	0	2	1	CC	CC		
244	3	0	0	1	1	CC	CC		
245	1	2	3	2	2	CC	CC	CC	CC
245	2	0	0	2	1	CC	CC		
245	3	0	0	1	1	CC	CC		
246	1	2	3	1	2	CC	CC	CC	CC
246	2	0	0	2	1	CC	CC		
246	3	0	0	1	1	CC	CC		
248	1	2	3	2	2	CC	CC	CC	CT
248	2	0	0	2	1	CC	CC		
248	3	0	0	1	1	CC	CT		
252	1	2	3	2	2	CC	CT	TT	CT
252	2	0	0	2	1	CT	TT		
252	3	0	0	1	1	CT	CC		
257	1	2	3	1	2	CT	CC	CT	CC
257	2	0	0	2	1	CT	CC		
257	3	0	0	1	1	CT	CC		
258	1	2	3	2	2	TT	CC	CT	CC
258	2	0	0	2	1	CT	CC		
258	3	0	0	1	1	TT	CC		
259	1	2	3	2	2	CC	TT	CC	CC
259	2	0	0	2	1	CC	CT		
259	3	0	0	1	1	CC	CT		
262	1	2	3		2	CC	CC	CC	CC
262	2	0	0	2	1	CC	CC		
262	3	0	0	1	1	CC	CC		
263	1	2	3		2	CC	CC	CC	CC
263	2	0	0	2	1	CC	CC		
263	3	0	0	1	1	CC	CC		
266	1	2	3		2	CT	CC	CT	CC
266	2	0	0	2	1	CC	CC		
266	3	0	0	1	1	TT	CC		
267	1	2	3		2	CC	CT	CT	CC
267	2	0	0	2	1	CT	CC		
267	3	0	0	1	1	CC	CT		
269	1	2	3		2	CT	CC	TT	CC
269	2	0	0	2	1	CT	CC		
269	3	0	0	1	1	TT	CC		
271	1	2	3		2	CT	CC	TT	CC
271	2	0	0	2	1	TT	CC		
271	3	0	0	1	1	CT	CC		
272	1	2	3		2	CT	CT	TT	CT
272	2	0	0	2	1	CT	CT		
272	3	0	0	1	1	TT	CT		
273	1	2	3		2	CT	CC	TT	CC
273	2	0	0	2	1	CT	CC		
273	3	0	0	1	1	TT	CC		
274	1	2	3		2	CC	CC	CC	CC
274	2	0	0	2	1	CC	CC		
274	3	0	0	1	1	CC	CC		
276	1	2	3		2	CC	CC	CT	CC
276	2	0	0	2	1	CT	CC		
276	3	0	0	1	1	CC	CC		

277	1	2	3	2	2	CC	CT	CT	CT
277	2	0	0	2	1	CT	CT		
277	3	0	0	1	1	CC	CT		
278	1	2	3	1	2	CT	CC	CT	CC
278	2	0	0	2	1	TT	CC		
278	3	0	0	1	1	CC	CC		
279	1	2	3		2	CT	CT	CT	CC
279	2	0	0	2	1	CT	CC		
279	3	0	0	1	1	CT	CT		
281	1	2	3	2	2	CT	CC	CC	CC
281	2	0	0	2	1	CT	CC		
281	3	0	0	1	1	CC	CC		
282	1	2	3		2	CC	CC	CT	CC
282	2	0	0	2	1	CC	CC		
282	3	0	0	1	1	CT	CC		
285	1	2	3		2	CC	CT	CT	CC
285	2	0	0	2	1	CT	CT		
285	3	0	0	1	1	CC	CC		
286	1	2	3		2	CT	CC	CC	CC
286	2	0	0	2	1	CT	CC		
286	3	0	0	1	1	CC	CC		
287	1	2	3		2	CC	CC	CT	CC
287	2	0	0	2	1	CC	CC		
287	3	0	0	1	1	CT	CC		
291	1	2	3		2	CC	CC	CC	CC
291	2	0	0	2	1	CC	CC		
291	3	0	0	1	1	CC	CC		
293	1	2	3		2	CC	CC	TT	CC
293	2	0	0	2	1	CT	CC		
293	3	0	0	1	1	CT	CC		
294	1	2	3		2	CC	CC	CC	CT
294	2	0	0	2	1	CC	CT		
294	3	0	0	1	1	CC	CC		
295	1	2	3		2	CT	TT	CC	CT
295	2	0	0	2	1	CC	TT		
295	3	0	0	1	1	CT	CT		
296	1	2	3	1	2	CC	CT	CC	CC
296	2	0	0	2	1	CC	CT		
296	3	0	0	1	1	CC	CC		
297	1	2	3	1	2	CC	CC	CT	CC
297	2	0	0	2	1	CT	CC		
297	3	0	0	1	1	CC	CC		
298	1	2	3	1	2	CC	CC	CC	CT
298	2	0	0	2	1	CC	CT		
298	3	0	0	1	1	CC	CC		
299	1	2	3	1	2	CC	CC	CC	CT
299	2	0	0	2	1	CC	CC		
299	3	0	0	1	1	CC	CT		
300	1	2	3		2	CT	CC	CC	CT
300	2	0	0	2	1	CC	CC		
300	3	0	0	1	1	CT	CT		
302	1	2	3	1	2	CT	CC	CC	CC
302	2	0	0	2	1	CC	CC		
302	3	0	0	1	1	CT	CC		
303	1	2	3	1	2	CT	CC	CT	CC
303	2	0	0	2	1	CT	CC		
303	3	0	0	1	1	CT	CC		
304	1	2	3		2	CC	CC	CC	CC
304	2	0	0	2	1	CC	CC		
304	3	0	0	1	1	CC	CC		
